# Mycophenolate mofetil modulates adhesion receptors of the beta1 integrin family on tumor cells: impact on tumor recurrence and malignancy

**DOI:** 10.1186/1471-2407-5-4

**Published:** 2005-01-11

**Authors:** Tobias Engl, Jasmina Makarević, Borna Relja, Iyad Natsheh, Iris Müller, Wolf-Dietrich Beecken, Dietger Jonas, Roman A Blaheta

**Affiliations:** 1 Zentrum der Chirurgie, Klinik für Urologie und Kinderurologie; Johann Wolfgang Goethe-Universität; Frankfurt am Main, Germany

## Abstract

**Background:**

Tumor development remains one of the major obstacles following organ transplantation. Immunosuppressive drugs such as cyclosporine and tacrolimus directly contribute to enhanced malignancy, whereas the influence of the novel compound mycophenolate mofetil (MMF) on tumor cell dissemination has not been explored. We therefore investigated the adhesion capacity of colon, pancreas, prostate and kidney carcinoma cell lines to endothelium, as well as their beta1 integrin expression profile before and after MMF treatment.

**Methods:**

Tumor cell adhesion to endothelial cell monolayers was evaluated in the presence of 0.1 and 1 μM MMF and compared to unstimulated controls. beta1 integrin analysis included alpha1beta1 (CD49a), alpha2beta1 (CD49b), alpha3beta1 (CD49c), alpha4beta1 (CD49d), alpha5beta1 (CD49e), and alpha6beta1 (CD49f) receptors, and was carried out by reverse transcriptase-polymerase chain reaction, confocal microscopy and flow cytometry.

**Results:**

Adhesion of the colon carcinoma cell line HT-29 was strongly reduced in the presence of 0.1 μM MMF. This effect was accompanied by down-regulation of alpha3beta1 and alpha6beta1 surface expression and of alpha3beta1 and alpha6beta1 coding mRNA. Adhesion of the prostate tumor cell line DU-145 was blocked dose-dependently by MMF. In contrast to MMF's effects on HT-29 cells, MMF dose-dependently up-regulated alpha1beta1, alpha2beta1, alpha3beta1, and alpha5beta1 on DU-145 tumor cell membranes.

**Conclusion:**

We conclude that MMF possesses distinct anti-tumoral properties, particularly in colon and prostate carcinoma cells. Adhesion blockage of HT-29 cells was due to the loss of alpha3beta1 and alpha6beta1 surface expression, which might contribute to a reduced invasive behaviour of this tumor entity. The enhancement of integrin beta1 subtypes observed in DU-145 cells possibly causes re-differentiation towards a low-invasive phenotype.

## Background

With the improved long-term outcome of allograft recipients in the cyclosporine or tacrolimus era, malignant tumors have become increasingly important. Malignant tumours develop in 15–20% of graft recipients after 10 years, and thus contribute substantially to the morbidity and mortality of these patients [1]. Malignancies can develop in three ways: de-novo occurrence in the recipient, recurrent malignancy in the recipient or transmission of malignancy from the donor. In all cases, the post-transplant treatment regimen and the level of immunosuppression are high risk factors due to the long-term modification of the immune system.

During the last years, the novel immunosuppressive drug mycophenolate mofetil (MMF) has been introduced into the clinical protocol to overcome severe side effects associated with cyclosporine or tacrolimus. Meanwhile, it has become part of the immunosuppressive regimen after liver, kidney or heart transplantation [2]. Still, the influence of MMF on tumor recurrence or de novo malignancy has not been explored.

MMF effects are based on the inhibition of inosine monophosphate dehydrogenase (IMPDH) and prevention of guanosine monophosphate synthesis from inosine monophosphate, a rate-limiting step in the purine biosynthesis in lymphocytes. Consequently, MMF blocks the proliferation and clonal expansion of T and B lymphocytes, and prevents the generation of cytotoxic T cells, as well as other effector T cells [3]. Additional mechanisms may also contribute to the efficacy of MMF in preventing allograft rejection. By depleting guanosine nucleotides, MMF suppresses glycosylation and the expression of some adhesion molecules, thereby decreasing the recruitment of lymphocytes and monocytes into sites of inflammation and graft rejection [3]. Immunoprecipitation studies have shown that one of the glycoproteins affected is the lymphocytic alpha4beta1 integrin, the ligand for VCAM-1 on activated endothelial cells. Further experiments have revealed inhibition of the integrin LFA-1, the counter-receptor of ICAM-1, after MMF administration [4,5].

The integrins constitute a family of transmembrane receptor proteins composed of heterodimeric complexes of noncovalently linked alpha and beta chains. Integrins function in cell-to-cell and cell-to-extracellular matrix (ECM) adhesive interactions and transduce signals from the ECM to the cell interior and vice versa. For various types of cancers, different changes in integrin expression are closely associated with tumor growth and metastasis. Based on the knowledge that MMF modulates integrin expression, we postulated that MMF might not only suppress leukocyte recruitment to the donor graft, but also prevent integrin-dependent tumor dissemination. To explore how far MMF might serve as a metastasis-blocking agent, we investigated the beta1 integrin subunit expression pattern of colon, kidney, pancreas and prostate tumor cells before and after MMF treatment, as well as MMF effects on tumor cell adhesion to human endothelium in vitro.

The present study indicates that MMF possesses anti-tumoral properties particularly to colon and prostate carcinoma cells. Alterations of the beta1 integrin profile are responsible for blocking tumor cell adhesion to vascular endothelium.

## Methods

### Cell cultures

Kidney carcinoma Caki I cells, pancreatic carcinoma DanG cells and colonic adenocarcinoma HT-29 cells G were obtained from the tumor cell bank of Johannes Gutenberg University, Mainz, Germany. Prostate carcinoma DU-145 cells were purchased from DSMZ (Braunschweig, Germany). Tumor cells were grown and subcultured in RPMI1640 medium (Seromed, Berlin, Germany) supplemented with 10% FCS, 100 IU/ml penicillin and 100 μg/ml streptomycin at 37°C in a humidified, 5% CO_2 _incubator.

Endothelial cells (HUVEC) were isolated from human umbilical veins and harvested by enzymatic treatment with chymotrypsin. HUVEC were grown in Medium 199 (Biozol, Munich, Germany), 10% fetal calf serum (FCS; Gibco, Karlsruhe, Germany), 10% pooled human serum (Blood Bank of The German Red Cross, Frankfurt am Main, Germany), 20 μg/ml endothelial cell growth factor (Boehringer, Mannheim, Germany), 0.1% heparin (Roche, Basel, Switzerland), 100 ng/ml gentamycin (Gibco) and 2% 1 M HEPES-buffer (Seromed, Berlin, Germany). To control the purity of HUVEC cultures, cells were stained with fluorescein isothiocyanate (FITC)- labelled monoclonal antibody against Factor VIII-associated antigen (Von Willebrand factor; clone F8/86; Dako, Hamburg, Germany) and analyzed microscopically or by FACscan (Becton Dickinson, Heidelberg, Germany; FL-1H (log) channel histogram analysis; 1 × 10^4 ^cells/scan). Cell cultures with a purity > 95% were serially passaged. Subcultures from passages 2–4 were selected for experimental use.

### Mycophenolate mofetil (MMF)

Tumor cells were pretreated with MMF (Roche Bioscience, Grenzach-Wyhlen, Germany) (0.1 μM, 1 μM). Before adding the MMF-treated tumor cells to HUVEC (monolayer adhesion assay), cell cultures were washed to remove MMF from the medium. Results were compared to untreated controls.

Viability of tumor cells in presence of MMF was assessed by propidium iodide dsDNA-intercalation or quantitative fluorescence analysis of enzyme-catalyzed fluorescein-diacetate metabolism.

### Monolayer adhesion assay

HUVEC were transferred to six-well multiplates (Falcon Primaria; Becton Dickinson, Heidelberg, Germany) in complete HUVEC-medium. When confluency was reached, 0.5 × 10^6 ^tumor cells of each entity/well were carefully added to the HUVEC monolayer for 60 min. Subsequently, non-adherent tumor cells were washed off using warmed (37°C) Medium 199. The adherent cells were fixed with 1% glutaraldehyde and counted in five different fields (5 × 0.25 mm^2^) using a phase contrast microscope (20 × objective) to calculate the mean cellular adhesion rate.

### Evaluation of integrin surface expression

Tumor cells were washed in blocking solution (PBS, 0.5% BSA) and then incubated for 60 min at 4°C with the FITC-conjugated monoclonal antibody anti-alpha2beta1 (Becton Dickinson; clone AK-7), anti-alpha4beta1 (Cymbus Biotechnology, Hofheim, Germany; clone HP2I1), anti-alpha5beta1 (Cymbus Biotechnology; clone SAM-1), anti-alpha6beta1 (Becton Dickinson; clone GOH3), or with the PE-conjugated monoclonal antibody anti-alpha1beta1 (Becton Dickinson; clone SR84), or anti-alpha3beta1 (Becton Dickinson; clone C3II1). Integrin expression of tumor cells was then measured using a FACscan (Becton Dickinson; FL-1H (log) channel histogram analysis; 1 × 10^4 ^cells/scan) and expressed as mean fluorescence units (MFU).

A mouse IgG1-FITC was used as an isotype control for FITC conjugated antibodies. To evaluate background staining of PE conjugated antibodies, goat anti mouse IgG-PE was used (all: Cymbus Biotechnology).

To analyze integrin beta1 distribution on the cell membrane, tumor cells were transferred to round cover slips (pretreated with 2% 3-aminopropyl-triethoxysilan) placed in a 24 well multiplate. Upon reaching confluency, cell cultures were washed and fixed in cold (-20°C) methanol/acetone (60/40 v/v). Subsequently, cells were incubated for 60 min with unconjugated anti-integrin monoclonal antibodies. Indocarbocyanine (Cy 3™; Dianova; working dilution: 1:50) conjugated goat-anti-mouse IgG was then added as the secondary antibody. To prevent photobleaching of the fluorescent dye, cover glasses with stained cells were taken out of the wells and the residual liquid was removed. These were then embedded in an antifade reagent / mounting medium mixture (ProLong™ Antifade Kit, MoBiTec, Göttingen, Germany) and mounted on slides. The slides were viewed using a confocal laser scanning microscope (LSM 10; Zeiss, Jena, Germany) with a plan-neofluar ×100 / 1.3 oil immersion objective.

### mRNA expression of beta1 integrins

mRNA expression of beta1 integrins was evaluated by reverse transcriptase-polymerase chain reaction (RT-PCR). Tumor cells were seeded in 50 ml culture flasks (25 cm^2 ^growth area; Falcon Primaria, Becton Dickinson) and cultured with or without MMF. Total RNA was extracted by using RNeasy kit (Qiagen, Hilden, Germany) and RNA samples were then treated with 80 U/ml of Rnase-free Dnase I (Boehringer Mannheim, Mannheim, Germany) for 60 min at 37°C, to eliminate amplifiable contaminating genomic DNA. Subsequently, samples were incubated for 10 min at 65°C to inactivate Dnase. Complementary DNA was synthesized from 1 μg of total RNA per sample with a 60 min incubation at 42°C, using the Moloney murine leukaemia virus reverse transcriptase (Invitrogen, Karlsruhe, Germany) and oligo-(dT) priming (Boehringer Mannheim). Amplification was carried out using gene specific primers and Platinum-Taq polymerase (Invitrogen) in a Mastercycler Gradient thermocycler (Eppendorf, Hamburg, Germany). Reactions were performed in the presence of 0.5 μl cDNA, with an initial incubation step at 95°C for 2 min. Cycling conditions consisted of denaturation at 95°C for 30 sec, annealing at 60°C for 30 sec and extension at 72°C for 30 sec over a total of 30 cycles. The reaction was completed by another 10 min incubation step at 72°C. The specific sequences for sense and anti-sense primers are shown in table 1. The PCR products were subjected to electrophoresis in 1.5% agarose gel and visualized by ethidium bromide.

### Statistical analysis

All studies were performed 3–6 times. Statistical significance was investigated by the Wilcoxon signed rank test showing two-sided probabilities and using normal approximation. Differences were considered statistically significant at a p value less than 0.05.

## Results

### MMF modulates tumor cell adhesion to HUVEC

**Figure 1:**
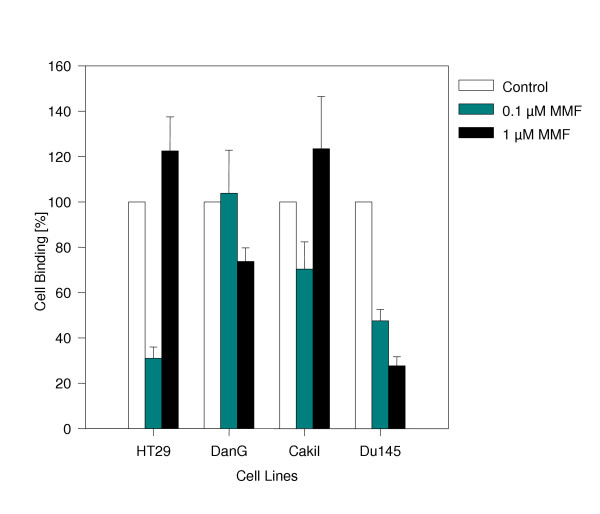
Adhesion capacity of HT-29, DanG, Caki I, and DU-145 tumor cells. Each tumor cell type was pretreated with 0.1 or 1 μM MMF or remained untreated (control) and was then added for 60 min to human umbilical vein endothelial cell monolayers. Non-adherent tumor cells were washed off in each sample, the remaining cells were fixed and counted in five different fields (5 × 0.25 mm^2^) using a phase contrast microscope. Adhesion capacity is depicted as tumor cell binding and related to 100% binding of non-treated control cell lines (mean ± SD; n = 6). Asterix indicates statistically significant difference to the control.

The 60 min adhesion rates of tumor cells were calculated at 22.5 ± 4.1 DanG cells/0.25 mm^2^, 39,8 ± 10.5 DU-145 cells/0.25 mm^2^, 55,3 ± 11.7 Caki I cells/0.25 mm^2^, or 80,5 ± 17.2 HT-29 cells/0.25 mm^2^. MMF differentially modulated the adhesive capacity of the tumor cells which was strongly dependent on the drug concentration and the cell line used (figure 1). Adhesion of DanG cells was weakly reduced by 1 μM MMF. A modest down-regulating effect was seen on Caki I cells at a MMF concentration of 0.1 μM. Strong and significant adhesion blockade was achieved when HT-29 cells were treated with 0.1 μM MMF (p = 0.0079). This effect was reverted at a dosage of 1 μM. Furthermore, MMF dose-dependently and significantly reduced the adhesive capacity of DU-145 cells with a maximum effect at 1 μM (p = 0.0079). In all experiments, cell viability was not impaired by MMF.

### beta1 integrin expression pattern

**Figure 2:**
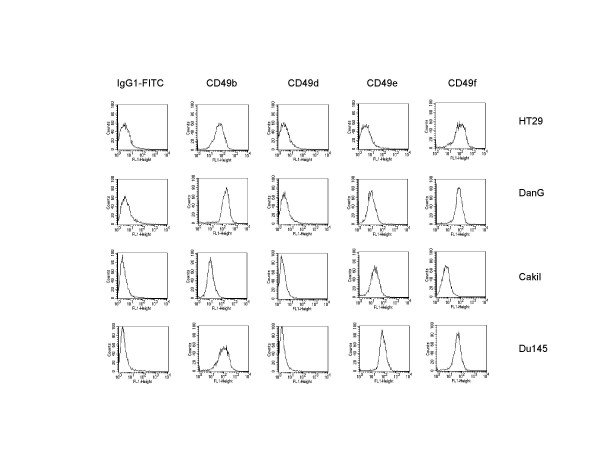
FACS analysis of beta1 integrin surface expression on HT-29, DanG, Caki I, and DU-145 tumor cells. Tumor cells were washed in blocking solution and then stained with the FITC-conjugated monoclonal antibody anti-alpha2beta1 (CD49b; clone AK-7), anti-alpha4beta1 (CD49d; clone HP2I1), anti-alpha5beta1 (CD49e; clone SAM-1), or anti-alpha6beta1 (CD49f; clone GOH3). A mouse IgG1-FITC was used as an isotype control for FITC conjugated antibodies. Fluorescence was analyzed using a FACScan flow cytometer, and a histogram plot (FL1-Height) was generated to show FITC-fluorescence.

**Figure 3:**
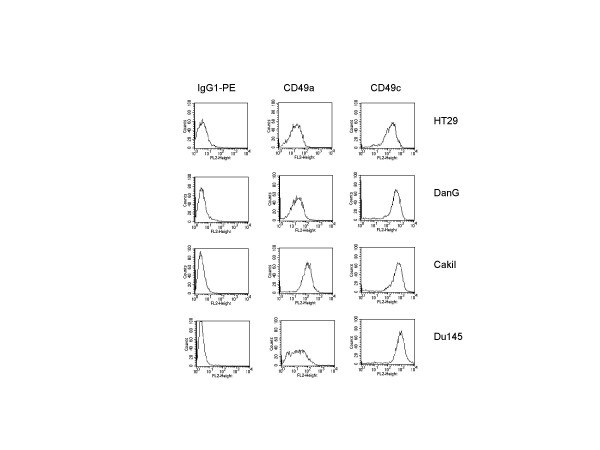
FACS analysis of beta1 integrin surface expression on HT-29, DanG, Caki I, and DU-145 tumor cells. Tumor cells were washed in blocking solution and then stained with the PE-conjugated monoclonal antibody anti-alpha1beta1 (CD49a; clone SR84), or anti-alpha3beta1 (CD49c; clone C3II1). To evaluate background staining of PE conjugated antibodies, goat anti mouse IgG-PE was used. Fluorescence was analyzed using a FACScan flow cytometer, and a histogram plot (FL1-Height) was generated to show FITC-fluorescence.

Figures 2 and 3 depict the integrin beta1 surface expression pattern on untreated tumor cell cultures. Figure 2 is related to FITC-labelled antibodies, figure 3 to PE-labelled antibodies. Each tumor entity was characterized by a specific integrin pattern. Integrins were expressed in the following order (MFU ± SD; n = 4):

DanG: alpha3beta1 (455.6 ± 71.0) > alpha2beta1 (175.7 ± 24.3) > alpha6beta1 (54.8 ± 9.2) > alpha1beta1 (27.3 ± 3.2).

Caki I: alpha3beta1 (601.3 ± 82.0) > alpha1beta1 (139.2 ± 24.0) > alpha5beta1 (22.9 ± 4.2).

HT-29: alpha3beta1 (202.0 ± 33.8) > alpha6beta1 (119.6 ± 14.5) > alpha2beta1 (69.2 ± 9.0) > alpha1beta1 (25.4 ± 3.4).

DU-145: alpha3beta1 (942.5 ± 112.9) > alpha2beta1 (95.4 ± 12.4) > alpha5beta1 (69.1 ± 8.9) > alpha6beta1 (50.9 ± 7.2) > alpha1beta1 (31.4 ± 4.8).

Mean IgG1-FITC isotype control was 8.6 ± 1.8 MFU, mean IgG1-PE isotype control was 9.9 ± 1.7 MFU.

Analysis of the mRNA expression level confirmed the flow cytometry data (see below).

**Figure 4:**
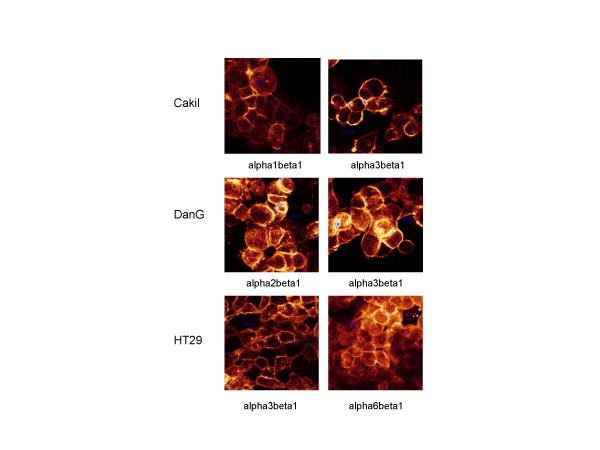
Confocal images of the distribution pattern of beta1 integrin receptor molecules on HT-29, DanG, and Caki I tumor cells. Integrin subtypes which were predominantly expressed on the respective tumor cell lines are shown. All: magnification × 100.

The distribution pattern of those integrins which were predominantly expressed on the respective tumor cell line was further explored by confocal microscopy (figure 4). alpha2beta1 (DanG cells), alpha3beta1 (Caki I, DanG HT-29 cells), and alpha6beta1 integrins (HT-29 cells) were distributed homogenously among the cell surface. In contrast, alpha1beta1 integrins on Caki I cells accumulated mainly at the sites of cell-cell-contacts.

### MMF modulates beta1 integrin surface expression

**Figure 5:**
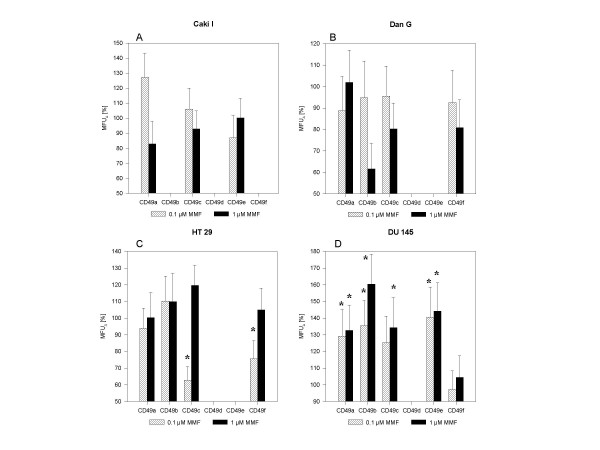
Dose-response analysis. Each tumor cell type was pretreated with 0.1 or 1 μM MMF and compared to untreated controls. beta1 integrin surface expression was evaluated by a FACscan using FITC-conjugated monoclonal antibody anti-alpha2beta1 (CD49b), anti-alpha4beta1 (CD49d), anti-alpha5beta1 (cd49e), anti-alpha6beta1 (CD49f), or PE-conjugated monoclonal antibody anti-alpha1beta1 (CD49a) or anti-alpha3beta1 (CD49c). A mouse IgG1-FITC or IgG-PE was used as isotype controls. Mean fluorescence values (MFU) of MMF treated cell cultures are related to non-treated controls which were set to 100% (MFU ± SD, n = 6). Asterix indicates statistically significant difference to the control.

MMF evoked distinct alterations of the beta1 integrin expression pattern (figure 5). MMF only slightly changed alpha1beta1, alpha3beta1, and alpha5beta1 integrin surface levels on Caki I cells (figure 5A), and weakly down-regulated alpha2beta1 on DanG cells when applied at 1 μM (figure 5B). However, 0.1 μM MMF strongly and significantly diminished alpha3beta1 (p = 0.0022) and alpha6beta1 integrins (p = 0.035) on HT-29 cells (figure 5C). This effect was reverted at concentrations of 1 μM MMF. The alpha3beta1 receptor became even slightly enhanced, compared to control values. alpha1beta1, alpha2beta1, alpha3beta1, alpha5beta1 on DU-145 cells were up-regulated significantly by MMF in a dose-dependent fashion, whereby strongest effects were seen on alpha2beta1 surface level in the presence of 1 μM MMF (>70% fluorescence enhancement, compared to non-treated controls; p = 0.0022; figure 5D).

### Influence of MMF on beta1 integrin coding mRNA

**Figure 6:**
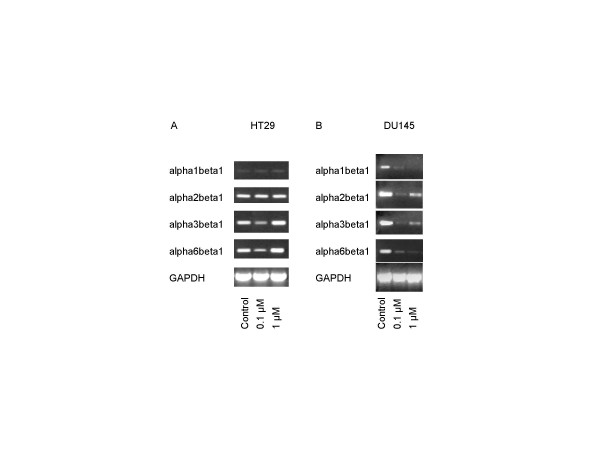
Analysis of beta1 integrin subtypes mRNA levels in HT-29 and DU-145 tumor cell lines. HUVEC were either grown in standard medium or in medium enriched with 0.1 or 1 μM MMF. mRNA expression level was investigated by reverse transcriptase-polymerase chain reaction. The housekeeping gene GAPDH served as the internal control.

To allow a clear interpretation of the strong effects of MMF on adhesion and beta1 integrin surface expression of HT-29 and DU-145 cells, MMF evoked alterations of gene activity was also evaluated in these cell lines (figure 6). Control experiments using non-treated HT-29 cells revealed high alpha2beta1, alpha3beta1, alpha6beta1 mRNA expression level (figure 6A). Application of 0.1 μM MMF induced down-regulation of alpha3beta1 and alpha6beta1 coding mRNA, which paralleled MMF's influence on receptor surface expression. The effect was reverted at a dosage of 1 μM. alpha3beta1 and alpha6beta1 coding mRNA became even slightly enhanced, compared to control experiments. With respect to the prostate tumor cell line DU-145, alpha1beta1, alpha2beta1, alpha3beta1 and alpha6beta1 coding mRNA was clearly detected in untreated cell cultures. Both, 0.1 μM and 1 μM MMF reduced mRNA of beta1 integrin subtypes, although the effect was more pronounced in the presence of 0.1 μM MMF (figure 6B).

## Discussion

Although MMF has become part of the standard regimen after organ transplantation its impact on tumor development and dissemination is still not clear.

Our adhesion experiments demonstrate that MMF down-regulates binding of tumor cells to endothelium, which might argue for anti-tumoral properties of this compound. Notably, HT-29 and DU-145 cells responded well to MMF (-70% adhesion reduction), while Caki I and DanG tumor cells were influenced only modestly. From a clinical viewpoint, distinct adhesion-blocking properties of MMF might be limited to colon and prostate carcinoma cells. Interestingly, HT-29 cells were more susceptible to MMF than DU-145 cells: 0.1 μM MMF was sufficient to significantly diminish adhesion of HT-29 cells, whereas 1 μM MMF was necessary to evoke maximum effects on DU-145 cells. The different sensitivity of the tumor cell lines to MMF might be caused by an unequal metabolic activity, coupled with variable IMPDH levels. Recent data have shown that the level of expression of IMPDH mRNA and protein differ among several cell lines [6], and that IMPDH is selectively up-regulated in neoplastic and replicating cells [7]. Although this has not yet been proven, MMF might be more effective in rapidly proliferating tumor cells than in tumors with a lower replicating activity. In this context, the average doubling times of HT-29 and DU-145 cultures during their exponential growth phase were calculated to be 13–16 h or 22 h, respectively [8-10], whereas the mean population doubling times of renal or pancreatic carcinoma cell lines ranged between 24–104 h or 16–40 h, respectively [11-14].

It should also be considered that MMF might switch on/off different intracellular signaling cascades in colon versus prostate tumor cells. Indeed, adhesion blockade of HT-29 cells was accompanied by reduced alpha3beta1 and alpha6beta1 surface expression, while adhesion blockade of DU-145 cells was accompanied by a dose-dependent up-regulation of integrins alpha1beta1, alpha2beta1, alpha3beta1, alpha5beta1 on the cell membrane.

Studies on integrin receptors presented evidence that beta1 integrin expression by colon carcinoma cells qualifies these cells to successfully adhere to secondary sites. Recent experiments have demonstrated that colon cancer cells adhere to endothelial cells via beta1 integrins and that addition of beta1 integrin blocking antibodies reduces tumor cell adhesion [15,16]. Based on a murine spleen injection-liver metastasis protocol, the alpha3beta1 integrin subtype was identified to predominantly facilitate the metastatic activity of colon cancer cells [17].

A converse scenario might be created during prostate carcinogenesis, as levels of beta1 integrins have been found reduced in neoplastic versus normal prostate tissue [18,19], and in malignant versus non-tumorigenic prostate cell lines [20]. An in vitro cell culture model revealed that TGF-beta stimulates the expression of alpha2beta1 integrin on prostate cancer cell lines and concomitantly reduces tumor cell adhesion to human bone marrow endothelium [21]. Down-regulation in the expression of the alpha3beta1 integrins may also allow prostate tumor cells to become more invasive and lead to an increased propensity for metastasis: When human alpha3beta1^high ^and alpha3beta1^low ^expressing prostate carcinoma cells were injected into immunocompromised SCID mice, only those cells with a drastically reduced integrin level were found to form tumors at the primary sites and to be highly invasive and metastatic [22]. This is in context with our data demonstrating beta1 integrin elevation on DU-145 prostate tumor cells in the context with diminished adhesion behaviour.

When discussing the relevance of integrins in tumor recurrence and malignancy, we should keep in mind that integrin receptors serve as mechanistic binding as well as differentiation triggering elements. Therefore, up-regulation of the same integrin type might either lead to enhanced cell adhesion by coupling the receptor to its ligand, or to a reduced cell adhesion by activating integrin driven differentiation signals. Based upon our in vitro assay, we conclude that MMF blocks adhesion of colon and prostate carcinoma cells by two different mechanisms: a) Loss of alpha3beta1 and alpha6beta1 surface expression directly contributes to the reduced adhesive behaviour of HT-29 cells, b) Enhancement of integrin beta1 subtypes might cause re-differentiation of DU-145 cells towards a low-adhesive phenotype. However, it still remains to be determined if MMF indeed acts as a differentiation inducing drug in prostate tumor cells. Beside the hypothesis that beta1 upregulation might activate differentiation inducing signals, selective inhibition of tumor-promoting pathways should also taken into consideration.

Presumably, down-regulation of alpha3beta1 and alpha6beta1 surface expression on HT-29 tumor cells might be caused by inhibition of receptor glycosylation and/or receptor de novo synthesis. The latter hypothesis seems to be more likely because MMF's effects at the cell surface were also observed at the mRNA level. This was not the case with DU-145 cells where MMF evoked up-regulation of membranous beta1 integrins was not paralleled by similar modifications of the beta1 integrin coding mRNA. There is still no clear concept why MMF causes integrin up-regulation in one tumor entity but down-regulation in another entity, both coupled with reduced tumor cell adhesiveness. Presumably, HT-29 and DU-145 tumor cells might be equipped with different enzyme systems, the intracellular signaling cascade might be activated differentially in colon versus prostate tumor cells, or sensitivity of specific pathways to MMF might differ between both tumor types.

Speculatively, alterations of post-translational events might change the receptor surface presentation in prostate carcinoma cells. Elegant experiments by Liang and coworkers demonstrated that over-expression of alpha5beta1 or beta1 integrin induced the decrease of protein kinase B (PKB) phosphorylation and subsequent accumulation of cyclin-dependent kinase inhibitor p21 [23]. A yeast-based two-hybrid system was employed which identified IMPDH as specifically interacting with PKB [24]. Furthermore, MMF treatment significantly increased p21 proteins, which could be reversed by the simultaneous addition of guanine or guanosine [25,26]. Hypothetically, p21 may act as an MMF triggered upstream signal (via PKB?), which contributes to enhanced beta1 integrin surface expression.

## Conclusions

The present study indicates that MMF possesses anti-tumoral properties particularly to colon and prostate carcinoma cells. Alterations of the beta1 integrin profile are responsible for blocking tumor cell adhesion to vascular endothelium. MMF might also act on further adhesion proteins which are relevant for tumor recurrence and dissemination. An in vitro study published recently refers to the sLeX-selectin pathway targeted by MMF [27]. CD44 glycoproteins as well as receptors of the cadherin family might also be modulated under MMF-based immunosuppressive regimen. From a clinical viewpoint, further studies must be undertaken which evaluate the tumor recurrence rate and classify the tumor type in MMF versus non-MMF treated transplant patients.

## Competing interests

The author(s) declare that they have no competing interests.

## Authors' contributions

TE performed parts of the in vitro studies, contributed toward the design of the study and drafted the manuscript. JM carried out confocal microscopy, BR and IN performed FACS-analyses. IM designed PCR primers and carried out the PCR studies. WDB contributed to the manuscript design and finalisation. DJ participated in the conception and design of the study. RAB carried out the adhesion assays, participated in the conception and design of the study and its coordination. All authors read and approved the final manuscript.

## Pre-publication history

The pre-publication history for this paper can be accessed here:



## Figures and Tables

**Table 1:** The sequences of the primers used for RT-PCR

mRNA	Sense primer sequence	Antisense primer sequence	bp
GAPDH	atcttccaggagcgagatcc	accactgacacgttggcagt	509
alpha1beta1	catgcgctcgttttggaa	cggccacatctcgggaccaga	309
alpha2beta1	gcatctcagaagtctgttgcc	cctgttgttaccttcagggag	335
alpha3beta1	tacgtgcgaggcaatgaccta	tttgggggtgcaggatgaagct	306
alpha6beta1	tggaggtacagttgttggcg	ctccgttaggttcagggagt	253
